# An Optimized Schwarz Method for the Optical Response Model Discretized by HDG Method

**DOI:** 10.3390/e25040693

**Published:** 2023-04-19

**Authors:** Jia-Fen Chen, Xian-Ming Gu, Liang Li, Ping Zhou

**Affiliations:** 1 School of Mathematics and Computer Science, Chongqing College of International Business and Economics, Chongqing 401520, China; 2 School of Mathematics, Southwestern University of Finance and Economics, Chengdu 611130, China; 3 School of Mathematical Sciences, University of Electronic Science and Technology of China, Chengdu 611731, China

**Keywords:** local optical response model, hybridizable discontinuous Galerkin method, optimized Schwarz method, Krylov subspace method, domain decomposition method

## Abstract

An optimized Schwarz domain decomposition method (DDM) for solving the local optical response model (LORM) is proposed in this paper. We introduce a hybridizable discontinuous Galerkin (HDG) scheme for the discretization of such a model problem based on a triangular mesh of the computational domain. The discretized linear system of the HDG method on each subdomain is solved by a sparse direct solver. The solution of the interface linear system in the domain decomposition framework is accelerated by a Krylov subspace method. We study the spectral radius of the iteration matrix of the Schwarz method for the LORM problems, and thus propose an optimized parameter for the transmission condition, which is different from that for the classical electromagnetic problems. The numerical results show that the proposed method is effective.

## 1. Introduction

Nanophotonics or nano-optics [1] is the study of the behavior of light on the nanometer scale, and of the interaction of nanometer-scale objects with light. Moreover, such a discipline is reshaping our worldview in many ways with fascinating (potential) applications such as novel biological detection and new storage media [2]. These applications require fine control of the propagation of light waves. So, it is important to use appropriate mathematical models to describe the behavior of the light–matter interactions [3]. Light waves are regarded as electromagnetic (EM) waves and modeled by Maxwell’s equations. Classical or semi-classical models can be employed to model the light–matter interactions, such as the Drude model, the hydrodynamic Drude model [4], and the nonlocal optical response model [5]. In this paper, we consider the classical local Drude model which is a fairly simple yet efficient oscillator model for free electrons in metals that performs well when the size of the considered nanostructure increases beyond ≥50 nm [6,7,8,9].

For a frequency–domain simulation with the Drude model, we are indeed required to solve the time-harmonic Maxwell’s equations whose closed-form solutions are not available. Thus, various numerical methods, such as the finite element (FE) method, discontinuous Galerkin (DG) method [10,11,12], and hybridizable discontinuous Galerkin (HDG) method, have been developed to solve Maxwell’s equations. Discretization by either an FE method [13] or an HDG method [14,15,16] can yield a large sparse discretized linear system. It is still difficult to solve the resulting system of linear algebraic equations by either a direct solver or a standard preconditioned iterative method. On the other hand, the domain decomposition method (DDM) of Schwarz-type is considered to be one of the most efficient solving strategies for Helmholtz-type problems and then has been extended for the time-harmonic Maxwell’s equations in [17,18,19]. Moreover, DDMs should be very suitable for implementing the high-performance parallel computations, because they can decompose the large-scale and complex boundary value problems (BVPs) into a series of small-scale and simple BVPs that can be solved separately. In short, DDMs are usually employed to deal with such large-scale problems [20]. In [17], where the permittivity is a real number, an optimized Schwarz method combined with an HDG method discretization was used for EM problems, and the coupling between the Schwarz method and the HDG method was shown to be natural.

In this paper, the permittivity is a complex number in the Drude model, which adds the complexity to the optimized Schwarz method. We derive an optimized transmission condition and a formulation of the spectral radius of the iteration matrix of the Schwarz method. Furthermore, the parameters for an optimized transmission condition are discussed and tested. The subdomain problems are discretized by an HDG method [16] and the resulting linear systems can be solved by a sparse direct solver. The popular Krylov subspace method, namely GMRES [21], is considered to accelerate the solution of the interface linear system.

There are five sections in the rest of this paper. First of all, the Drude model and some notations are briefly introduced in Section 2. The discretization of Drude model by an HDG formulation are described in Section 3. In Section 4, we present the formulations of a Schwarz algorithm and study the parameters of an optimized transmission condition in a two subdomains setting. Numerical tests are presented in Section 5 to show the effectiveness of the proposed method. Finally, we draw some concluding remarks in Section 6.

## 2. Problem and Notations

In this section, we will introduce several concepts and notations which are essential for our present study.

### 2.1. Maxwell’s Equations with Drude Model

We consider the 2D Maxwell’s equations in the frequency domain with a first-order Silver–Müller absorbing boundary condition (i.e., an artificial absorbing boundary condition) [22]

(1) $$\left\{\begin{array}{c} \mathrm{Curl}H=-i\omega \varepsilon_{0}\varepsilon E,\;\;\;\;\;\;\;\;\;\;\;\;\;\;\;\;\;\;\;\;\;\;\;\;\;\mathrm{in}\;\;\Omega ,\\ \mathrm{curl}E=i\omega \mu_{0}H,\;\;\;\;\;\;\;\;\;\;\;\;\;\;\;\;\;\;\;\;\;\;\;\;\;\;\;\;\;\;\mathrm{in}\;\;\Omega ,\\ n\times E-H=n\times E^{\mathrm{inc}}-H^{\mathrm{inc}}=g^{\mathrm{inc}},\;\;\mathrm{on}\;\Gamma_{a}, \end{array}\right.$$

where $i=\sqrt{-1}$ stands for the imaginary unit, ω refers to the angular frequency of the light wave, ε, $\varepsilon_{0}$, and $\mu_{0}$ represent the relative permittivity, the permittivity of free space, and the permeability of free space, respectively, $E=\left(\begin{array}{c} E_{x},E_{y} \end{array}\right)$ and $H=H_{z}$ denote the electric and magnetic fields, the superscript “$\cdot^{\mathrm{inc}}$” means the incident field, and n is the outward unit normal vector. The differential operators in this 2D setting are $\mathrm{Curl}H=(\partial_{y}H,-\partial_{x}H)$ and $\mathrm{curl}E=\partial_{x}E_{y}-\partial_{y}E_{x}$. The computational domain is denoted by Ω, and the artificial absorbing boundary is denoted by $\Gamma_{a}$ [22].

Note that ε=1 in the free space. According to the Drude model, ε accounts for the interactions between the time-varying electric field and the electron gas [2]. It varies with the angular frequency of the incoming light, i.e.,

(2) $$\varepsilon \left(\omega \right)=1-\frac{\omega_{p}^{2}}{\omega (\omega +i\gamma )},$$

where $\omega_{p}$ denotes the bulk plasma frequency of the material and γ is a damping constant. In [23], the authors consider the time-domain Maxwell–Drude model with a DG time-domain method.

### 2.2. Notations

We write here a triangulation $T_{h}$ of Ω with *K* denoting an element of discrete mesh. $F_{h}$, $F_{h}^{I}$, and $F_{h}^{B}$ represent the set of all edges of $T_{h}$, the set of all the edges of $T_{h}$ associated with the nanostructure, and the union of all the boundary edges of $T_{h}$, respectively. For an element $K^{1}\in T_{h}$ and its adjacent element $K^{2}$, $F=K^{1}⋂K^{2}$ is the common edge of $K^{1}$ and $K^{2}$. Let $\left(v^{1},v^{1}\right)$ be the traces of (v,v) on *F* from the interior of $K^{1}$ and $\left(v^{2},v^{2}\right)$ be the traces of (v,v) on *F* from the interior of $K^{2}$. $n^{1,2}$ and $t^{1,2}$ stand for the outward unit normal vectors to $K^{1,2}$ and the unit tangent vectors to the boundaries $\partial K^{1,2}$, respectively, so we have $t^{1}\times n^{1}=1$ and $t^{2}\times n^{2}=1$. On the face *F*, {·} and 〚·〛 can be defined as

$$\left\{\begin{array}{l} {\left\{v\right\}}_{F}=\frac{1}{2}\left(v^{1}+v^{2}\right),\\ {\left\{v\right\}}_{F}=\frac{1}{2}\left(v^{1}+v^{2}\right),\\ 〚n\times v{〛}_{F}=n^{1}\times v^{1}+n^{2}\times v^{2},\\ 〚vt{〛}_{F}=v^{1}t^{1}+v^{2}t^{2}. \end{array}\right.$$

For each $K\in T_{h}$ ($F\in F_{h}$), p≥0 refers to the local interpolation order, and $P_{p}\left(K\right)$ ($P_{p}\left(F\right)$) refers to the space of polynomial functions of degree at most *p*. We define the discontinuous FE spaces $V_{h}^{p}$, $V_{h}^{p}$, and a traced FE space $M_{h}^{p}$ as follows

$$V_{h}^{p}=\{v\in L^{2}{\left(\Omega \right)|v|}_{K}\in P_{p}\left(K\right),\;\forall K\in T_{h}\},$$

$$V_{h}^{p}=\{v\in {\left(L^{2}\left(\Omega \right)\right)}^{2}{\left|v\right|}_{K}\in {\left(P_{p}\left(K\right)\right)}^{2},\;\forall K\in T_{h}\},$$

$$M_{h}^{p}=\{\eta \in L^{2}\left(F_{h}\right){\left|\eta \right|}_{F}\in P_{p}\left(F\right),\;\forall F\in F_{h}\;\mathrm{and}\;\eta \left|\Gamma_{m}=0\right\}.$$

Note that $L^{2}\left(\Omega \right)$ represents the space of a squared integrable functions over Ω, where $\Gamma_{m}$ satisfies $\Gamma_{m}\cup \Gamma_{a}=\partial \Omega$, $\Gamma_{m}\cap \Gamma_{a}=⌀$. Note that ∂Ω denotes the boundary. $M_{h}^{p}$ consists of the functions which are not continuous at its ends, but continuous on an edge. For a domain *D* in $R^{2}$, u, v in ${\left(L^{2}\left(D\right)\right)}^{2}$ and *u*, *v* in $L^{2}\left(D\right)$, ${\left(u,v\right)}_{D}\;\mathrm{refers}\;\mathrm{to}\int_{D}u\cdot \bar{v}dx$ where $\bar{\cdot }$ denotes the complex conjugation and ${\left(u,v\right)}_{D}$ stands for the inner product $\int_{D}u\bar{v}dx$. On an interface *F*, ${\langle u,v\rangle }_{F}$ stands for the inner product $\int_{F}u\bar{v}\;ds$. So on the whole domain Ω, we have

$${\left(\cdot ,\cdot \right)}_{T_{h}}=\sum_{K\in T_{h}}{\left(\cdot ,\cdot \right)}_{K},\;\;{\langle \cdot ,\cdot \rangle }_{\partial T_{h}}=\sum_{K\in T_{h}}{\langle \cdot ,\cdot \rangle }_{\partial K},$$

$$\;\;{\langle \cdot ,\cdot \rangle }_{F_{h}}=\sum_{F\in F_{h}}{\langle \cdot ,\;\;\cdot \rangle }_{F},\;\;{\langle \cdot ,\cdot \rangle }_{\Gamma_{a}}=\sum_{F\in F_{h}⋂\Gamma_{a}}{\langle \cdot ,\cdot \rangle }_{F}.$$

## 3. HDG Formulations

We consider an approximate solution $\left(E_{h},H_{h}\right)$ of 2D Maxwell’s equations in the space $V_{h}^{p}\times V_{h}^{p}$ that satisfies for each element *K*

$$\left\{\begin{array}{c} {\left(i\omega \varepsilon_{0}\varepsilon E_{h},v\right)}_{K}+{\left(\mathrm{Curl}H_{h},v\right)}_{K}=0,\;\;\;\;\;\;\;\forall v\in V^{p}\left(K\right),\\ {\left(\mathrm{curl}E_{h},v\right)}_{K}-{\left(i\omega \mu_{0}H_{h},v\right)}_{K}=0,\;\;\;\;\;\;\;\;\;\;\;\forall v\in V^{p}\left(K\right). \end{array}\right.$$

Use the Green’s formula for the above equations and replace the boundary terms with the numerical traces ${\hat{E}}_{h}$, ${\hat{H}}_{h}$. One can have

(3) $$\left\{\begin{array}{l} {\left(i\omega \varepsilon_{0}\varepsilon E_{h},v\right)}_{K}+{\left(H_{h},\mathrm{curl}v\right)}_{K}-{\langle {\hat{H}}_{h},n\times v\rangle }_{\partial K}=0,\;\;\;\;\;\;\;\forall v\in V^{p}\left(K\right),\\ (E_{h},{\mathrm{Curl}v)}_{K}+{\langle n\times {\hat{E}}_{h},v\rangle }_{\partial K}-{\left(i\omega \mu_{0}H_{h},v\right)}_{K}=0,\;\;\;\;\;\;\;\;\forall v\in V^{p}\left(K\right). \end{array}\right.$$

A proper choice of numerical trace ${\hat{E}}_{h}$, ${\hat{H}}_{h}$ affects the correctness and the convergence of the discrete problem (3). According to the ideas in [16], we choose a hybrid variable $\lambda_{h}\in M_{h}^{p}$, and set ${\hat{E}}_{h}$ and ${\hat{H}}_{h}$ as follows

(4) $$\left\{\begin{array}{l} {\hat{H}}_{h}=\lambda_{h},\\ {\hat{E}}_{h}=E_{h}+\tau \left(H_{h}-\lambda_{h}\right)t, \end{array}\right.$$

where τ>0 is the local stabilization parameter. Considering the contributions of Equation (3) over all elements, the artificial absorbing boundary condition in the formulation of this conservativity condition and enforcing the continuity of the tangential component of ${\hat{E}}_{h}$, we have

(5) $$\left\{\begin{array}{l} {\left(i\omega \varepsilon_{0}\varepsilon E_{h},v\right)}_{T_{h}}+{\left(H_{h},\mathrm{curl}v\right)}_{T_{h}}-{\langle \lambda_{h},n\times v\rangle }_{\partial T_{h}}=0,\;\;\;\;\;\;\;\;\;\;\;\;\;\;\;\;\forall v\in V^{p}\left(K\right),\\ {\left(\mathrm{curl}E_{h},v\right)}_{T_{h}}-{\langle \tau \left(H_{h}-\lambda_{h}\right),v\rangle }_{\partial T_{h}}-{\left(i\omega \mu_{0}H_{h},v\right)}_{T_{h}}=0,\;\;\;\;\;\;\;\;\;\;\;\;\forall v\in V^{p}\left(K\right),\\ {\langle n\times E_{h},\eta \rangle }_{\partial T_{h}}-{\langle \tau \left(H_{h}-\lambda_{h}\right),\eta \rangle }_{\partial T_{h}}-{\langle \lambda_{h},\eta \rangle }_{\Gamma_{a}}={\langle g^{\mathrm{inc}},\eta \rangle }_{\Gamma_{a}},\;\;\;\;\;\;\;\;\forall \eta \in M_{h}^{p}. \end{array}\right.$$

Using the variable $\lambda_{h}$ to express $E_{h}$ and $H_{h}$, then we can obtain a global problem with only the unknown $\lambda_{h}$

(6) $$a_{h}\left(\lambda_{h},\eta \right)=b_{h}\left(\eta \right),$$

since all the interior faces satisfy the conservativity condition, we have

(7) $${\langle 〚n\times {\hat{E}}_{h}〛,\eta \rangle \;}_{F_{h}^{I}}=0,$$

and inserting ${\hat{E}}_{h}=E_{h}+\tau \left(H_{h}-\lambda_{h}\right)t$ into Equation (7), we can obtain

(8) $$\begin{array}{cc} 〚n\times {\hat{E}}_{h}〛\; & =〚n\times (E_{h}+\tau \left(H_{h}-\lambda_{h}\right)t)〛\\ & =〚n\times E_{h}〛-〚\tau \left(H_{h}-\lambda_{h}\right)〛\\ & =〚n\times E_{h}〛-\tau_{1}H_{h}^{1}-\tau_{2}H_{h}^{2}+\left(\tau_{1}+\tau_{2}\right)\lambda_{h}=0, \end{array}$$

where the superscript and the subscript 1 and 2 denote the values from the two elements coupled by edge. Therefore, the numerical traces can be expressed as

(9) $${\hat{H}}_{h}=\lambda_{h}=\frac{1}{\tau_{1}+\tau_{2}}\left(\tau_{1}H_{h}^{1}+\tau_{2}H_{h}^{2}\right)-\frac{1}{\tau_{1}+\tau_{2}}〚n\times E_{h}〛,$$

and using a similar method we also can obtain

(10) $${\hat{E}}_{h}=\frac{1}{\tau_{1}+\tau_{2}}\left(\tau_{2}E_{h}^{1}+\tau_{1}E_{h}^{2}\right)+\frac{\tau_{1}\tau_{2}}{\tau_{1}+\tau_{2}}〚H_{h}t〛.$$

## 4. An Optimized Schwarz Method

To introduce the Schwarz method, we divide the domain Ω into $\Omega_{1}$ and $\Omega_{2}$, and note that $\Omega_{1}$ and $\Omega_{2}$ are two non-overlapping subdomains. One can easily obtain the case that the domain Ω is divided into many subdomains, because the transmission condition only involves the adjacent subdomains. For the given initial guesses $(E^{l,0},H^{l,0}),\;l=1,2$, on the interface between the subdomains, we can compute $\left(E^{l,n+1},H^{l,n+1}\right)$ from $\left(E^{l,n},H^{l,n}\right)$ with the following Schwarz method [17].

(11) $$\begin{array}{c} \left\{\begin{array}{c} i\omega \varepsilon_{0}\varepsilon E^{1,n+1}+\mathrm{Curl}H^{1,n+1}=0,\;\;\;\;\;\;\;\;\;\;\;\;\;\;\;\;\;\;\;\;\mathrm{in}\;\Omega_{1},\\ \mathrm{curl}E^{1,n+1}-i\omega \mu_{0}H^{1,n+1}=0,\;\;\;\;\;\;\;\;\;\;\;\;\;\;\;\;\;\;\;\;\;\;\;\mathrm{in}\;\Omega_{1},\\ n\times E^{1,n+1}-H^{1,n+1}=n\times E^{\mathrm{inc}}-H^{\mathrm{inc}}=g^{\mathrm{inc}},\;\mathrm{on}\;\Gamma_{1a},\\ B_{n_{1}}\left(E^{1,n+1},H^{1,n+1}\right)=B_{n_{1}}\left(E^{2,n},H^{2,n}\right),\;\;\;\;\;\;\;\;\;\;\;\mathrm{on}\;\Gamma^{1}. \end{array}\right.\\ \left\{\begin{array}{c} i\omega \varepsilon_{0}\varepsilon E^{2,n+1}+\mathrm{Curl}H^{2,n+1}=0,\;\;\;\;\;\;\;\;\;\;\;\;\;\;\;\;\;\;\;\;\;\mathrm{in}\;\Omega_{2},\\ \mathrm{curl}E^{2,n+1}-i\omega \mu_{0}H^{2,n+1}=0,\;\;\;\;\;\;\;\;\;\;\;\;\;\;\;\;\;\;\;\;\;\;\;\;\mathrm{in}\;\Omega_{2},\\ n\times E^{2,n+1}-H^{2,n+1}=n\times E^{\mathrm{inc}}-H^{\mathrm{inc}}=g^{\mathrm{inc}},\;\;\mathrm{on}\;\Gamma_{2a},\\ B_{n_{2}}\left(E^{2,n+1},H^{2,n+1}\right)=B_{n_{2}}\left(E^{1,n},H^{1,n}\right),\;\;\;\;\;\;\;\;\;\;\;\;\mathrm{on}\;\Gamma^{2}. \end{array}\right. \end{array}$$

**Figure 1:**
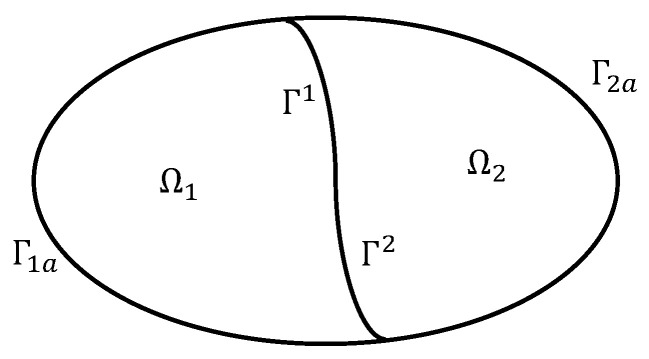
Some parameters on computational domain.

The computational domain Ω is displayed in Figure 1, where $\Gamma^{1,2}$ denotes the interface between the two adjacent subdomains. $\Gamma_{1a}$ and $\Gamma_{2a}$ denote the artificial absorbing boundary in each subdomain. The transmission condition is defined as $B_{n_{l}}\left(E,H\right)=S_{l}H+n\times E$, $\tau_{i}=S_{l},\;i,l=1,2,\;i\neq l$. In the following, we will show that the coupling between the Schwarz method and the HDG method is natural.

We set $K_{1}\in \bar{\Omega_{1}},K_{2}\in \bar{\Omega_{2}}$ to be two elements sharing a common face *F* between two adjacent subdomains. We denote $n_{1,2}$ as the outward unit normal vectors to $K_{1,2}$ and impose Dirichlet data $H_{h}^{1,n+1}=\hat{H_{h}^{1}}\left(H_{h}^{1,n+1},E_{h}^{1,n+1},H_{h}^{2,n},E_{h}^{2,n}\right)$ on *F* with $K_{1}$, then from Equation (9) we have

$$\tau_{2}H_{h}^{1,n+1}=\tau_{2}H_{h}^{2,n}-〚n\times E_{h}〛,$$

and using $〚n\times E_{h}〛=n_{1}\times E_{h}^{1,n+1}-n_{1}\times E_{h}^{2,n}$, so we have

$$\tau_{2}H_{h}^{1,n+1}=\tau_{2}H_{h}^{2,n}-\left(n_{1}\times E_{h}^{1,n+1}-n_{1}\times E_{h}^{2,n}\right),$$

that is

$$\tau_{2}H_{h}^{1,n+1}+n_{1}\times E_{h}^{1,n+1}=\tau_{2}H_{h}^{2,n}+n_{1}\times E_{h}^{2,n},$$

we can reach similar conclusions with $K_{2}$

$$\tau_{1}H_{h}^{2,n+1}+n_{2}\times E_{h}^{2,n+1}=\tau_{1}H_{h}^{1,n}+n_{2}\times E_{h}^{1,n}.$$

Then one can set

$$S_{1}=\tau_{2},\;S_{2}=\tau_{1},$$

and we have the transmission condition

(12) $$B_{n}\left(E,H\right)=S_{l}H+n\times E.\;$$

**Remark 1.**  *Notice when $S_{l}=\frac{1}{\sqrt{Re(\varepsilon )}},\;l=1,2$, the transmission condition will be the Silver–Müller condition, where Re(·) takes the real part of a complex number. We call it the classical transmission condition [17].*

### 4.1. Optimized Parameters for Optimized Schwarz Method

In the following, we try to give an analysis of the theoretical spectral radius of the iteration matrix of the Schwarz iteration, which is similar to that in [24]. Suppose that $\Omega_{1}$ is the left half plane (−∞,0]×(−∞,+∞) and $\Omega_{2}$ is the right half plane (0,+∞)×(−∞,+∞). Taking a Fourier transform in the *y* direction with Equation (1), we obtain

(13) $$\left\{\begin{array}{l} i\omega \varepsilon_{0}\varepsilon E_{x}-ikH=0,\\ i\omega \varepsilon_{0}\varepsilon E_{y}-\partial_{x}H=0,\\ \partial_{x}E_{y}+ikE_{x}-i\omega \mu_{0}H=0, \end{array}\right.$$

where *k* is the Fourier coefficient. According to the first equation of Equation (13), replacing $E_{x}$ with *H*, we obtain

(14) $$\partial_{x}\left[\begin{array}{c} H\\ E_{y} \end{array}\right]+\left[\begin{array}{cc} 0 & -i\omega \varepsilon_{0}\varepsilon \\ \frac{ik^{2}}{\omega \varepsilon_{0}\varepsilon }-i\omega \mu_{0} & 0 \end{array}\right]\left[\begin{array}{c} H\\ E_{y} \end{array}\right]=0.$$

Because of the radiation condition, the solution of Equation (14) in $\Omega_{l}$ (l=1,2) is given by

(15) $$\left[\begin{array}{c} H^{1}\\ E_{y}^{1} \end{array}\right]=a_{1}v_{1}e^{\lambda_{1}x}=a_{1}\left[\begin{array}{c} \frac{i\omega \varepsilon_{0}\varepsilon }{\lambda_{1}}\\ 1 \end{array}\right]e^{\lambda_{1}x},$$

(16) $$\left[\begin{array}{c} H^{2}\\ E_{y}^{2} \end{array}\right]=a_{2}v_{2}e^{\lambda_{2}x}=a_{2}\left[\begin{array}{c} \frac{i\omega \varepsilon_{0}\varepsilon }{\lambda_{2}}\\ 1 \end{array}\right]e^{\lambda_{2}x},$$

where $\lambda_{1}=\sqrt{k^{2}-\omega^{2}\varepsilon_{0}\mu_{0}\varepsilon }$, $\lambda_{2}=-\sqrt{k^{2}-\omega^{2}\varepsilon_{0}\mu_{0}\varepsilon }$ are the eigenvalues of the coefficient matrix in Equation (14), $v_{1}$, $v_{2}$ are their corresponding eigenvectors, and the coefficients $a_{1},a_{2}$ are uniquely determined by the transmission conditions. Set $\lambda =\lambda_{1}=-\lambda_{2}=\sqrt{k^{2}-\omega^{2}\varepsilon_{0}\mu_{0}\varepsilon }$. Inserting Equations (15) and (16) into the last equation of Equation (11), we have

$$\left\{\begin{array}{c} S_{1}a_{1}^{n+1}\frac{i\omega \varepsilon_{0}\varepsilon }{\lambda }e^{\lambda x}+a_{1}^{n+1}e^{\lambda x}=-S_{1}a_{2}^{n}\frac{i\omega \varepsilon_{0}\varepsilon }{\lambda }e^{-\lambda x}+a_{2}^{n}e^{-\lambda x},\\ -S_{2}a_{2}^{n+1}\frac{i\omega \varepsilon_{0}\varepsilon }{\lambda }e^{-\lambda x}-a_{2}^{n+1}e^{-\lambda x}=S_{2}a_{1}^{n}\frac{i\omega \varepsilon_{0}\varepsilon }{\lambda }e^{\lambda x}-a_{1}^{n}e^{\lambda x}. \end{array}\right.$$

At the *n*-th step of the Schwarz algorithm with x=0, the coefficients $a_{1},a_{2}$ satisfy the system

$$a_{1}^{n+1}=\frac{-S_{1}i\omega \varepsilon_{0}\varepsilon +\lambda }{S_{1}i\omega \varepsilon_{0}\varepsilon +\lambda }\frac{S_{2}i\omega \varepsilon_{0}\varepsilon -\lambda }{-S_{2}i\omega \varepsilon_{0}\varepsilon -\lambda }a_{1}^{n-1},$$

then we have the spectral radius ρ in the form

(17) $$\rho =\frac{-S_{1}i\omega \varepsilon_{0}\varepsilon +\sqrt{k^{2}-\omega^{2}\varepsilon_{0}\mu_{0}\varepsilon }}{S_{1}i\omega \varepsilon_{0}\varepsilon +\sqrt{k^{2}-\omega^{2}\varepsilon_{0}\mu_{0}\varepsilon }}\frac{S_{2}i\omega \varepsilon_{0}\varepsilon -\sqrt{k^{2}-\omega^{2}\varepsilon_{0}\mu_{0}\varepsilon }}{-S_{2}i\omega \varepsilon_{0}\varepsilon -\sqrt{k^{2}-\omega^{2}\varepsilon_{0}\mu_{0}\varepsilon }}.$$

In order to derive an optimized transmission condition, one can set the second-order approximation of the operator $S_{l}=\alpha_{l}+\beta_{l}\partial_{\tau }^{2}$, l=1,2, see Reference [25], where $\partial_{\tau }^{2}$ denotes the second-order derivative along with the interface and $\alpha_{l},\beta_{l}$ are the parameters to be determined [17]. With the zeroth order approximation of the operator $S_{l}$, i.e., $S_{1}=\alpha_{1},\;S_{2}=\alpha_{2}$, where $\alpha_{l}\;\left(l=1,2\right)$ are two complex numbers, then

(18) $$\rho =\frac{-\alpha_{1}i\omega \varepsilon_{0}\varepsilon +\sqrt{k^{2}-\omega^{2}\varepsilon_{0}\mu_{0}\varepsilon }}{\alpha_{1}i\omega \varepsilon_{0}\varepsilon +\sqrt{k^{2}-\omega^{2}\varepsilon_{0}\mu_{0}\varepsilon }}\frac{\alpha_{2}i\omega \varepsilon_{0}\varepsilon -\sqrt{k^{2}-\omega^{2}\varepsilon_{0}\mu_{0}\varepsilon }}{-\alpha_{2}i\omega \varepsilon_{0}\varepsilon -\sqrt{k^{2}-\omega^{2}\varepsilon_{0}\mu_{0}\varepsilon }}.$$

Therefore one can consider the optimization problem [24,25] as follows to determine the optimized parameters $\alpha_{l}\;\left(l=1,2\right)$

(19) $$\alpha_{l}^{*}=\arg\min_{\alpha_{1},\alpha_{2}}\left(\max\left(\left|\rho \right|\right)\right),\;l=1,2.$$

Unfortunately, it is difficult to solve this optimization problem explicitly because this problem is an open problem. For classic Maxwell’s equations with real permittivity, $\alpha_{l}={\left(i\omega \right)}^{\left(-1\right)}\left(p_{l}+ip_{l}\right)$ is often used [17,19,24], where

(20) $$p_{l}=\frac{\sqrt{\pi }C_{w}^{\frac{1}{4}}}{\sqrt{2}\sqrt{h}},\;\;\mathrm{and}\;\;C_{w}=\min\left(k_{1}^{2}-\omega^{2},\omega^{2}-k_{2}^{2}\right),$$

where $k_{1}$ and $k_{2}$ are the highest and lowest possible frequency allowed [24]. However, this choice does not work well for the Drude model. We present four possible guesses of $\alpha_{l}$ in Table 1 which lead to different optimized transmission conditions. The term “classical” in Table 1 represents classical transmission condition mentioned in Remark 1. Case3 is the above common choice.

As seen from Table 1, we find that the Schwarz methods with the last two cases do not converge at all. Case1 is theoretically the best choice which can be seen from the spectral radius ρ. In the next sections, numerical results will confirm this theoretical observation.

### 4.2. HDG Discretization

Let $\Gamma_{a}^{i}=\Gamma_{a}⋂\partial \Omega_{i}$ and $\Gamma_{i,j}=\partial \Omega_{i}⋂\Omega_{j},\;i,j=1,2$. Using the optimized transmission conditions on the interface $\Gamma_{i,j}$, one can make discrete Equation (11) using the HDG formulation Equation (5) which yields a problem as Equation (21). By finding $\left(E_{h}^{i,n+1},H^{i,n+1},\lambda_{h}^{i,n+1}\right)\left(n=1,2,\ldots \right)$ until convergence, and satisfying for all $v\in V_{h}^{i},\;v\in V_{h}^{i}$ and $\eta \in M_{h}^{i}$, we can obtain the solution

(21) $$\left\{\begin{array}{l} {\left(i\omega \varepsilon_{0}\left(1-\frac{\omega_{p}^{2}}{\omega (\omega +i\gamma )}\right)E_{h}^{i,n+1},v\right)}_{T_{h}^{i}}+{\left(H_{h}^{i,n+1},\mathrm{curl}v\right)}_{T_{h}^{i}}-{\langle \lambda_{h}^{i,n+1},n\times v\rangle }_{\partial T_{h}^{i}}=0,\\ {\left(\mathrm{curl}E_{h}^{i,n+1},v\right)}_{T_{h}^{i}}-{\langle \tau \left(H_{h}^{i,n+1}-\lambda_{h}^{i,n+1}\right),v\rangle }_{\partial T_{h}^{i}}-{\left(i\omega \mu_{0}H_{h}^{i,n+1},v\right)}_{T_{h}^{i}}=0,\\ {\langle S_{i}\lambda_{h}^{i,n+1},\eta \rangle }_{\Gamma_{i,j}}+{\langle n\times E_{h}^{i,n+1},\eta \rangle }_{\partial T_{h}^{i}}-{\langle \tau H_{h}^{i,n+1},\eta \rangle }_{\partial T_{h}^{i}}+{\langle \tau \lambda_{h}^{i,n+1},\eta \rangle }_{\partial T_{h}^{i}}\\ -{\langle \lambda_{h}^{i,n+1},\eta \rangle }_{\Gamma_{a}^{i}}={\langle S_{i}\lambda_{h}^{j,n},\eta \rangle }_{\Gamma_{i,j}}+{\langle n\times E_{h}^{j,n},\eta \rangle }_{\partial T_{h}^{j}}-{\langle \tau H_{h}^{j,n},\eta \rangle }_{\partial T_{h}^{j}}\\ +{\langle \tau \lambda_{h}^{j,n},\eta \rangle }_{\partial T_{h}^{j}}+{\langle g^{\mathrm{inc}},\eta \rangle }_{\Gamma_{a}^{i}}, \end{array}\right.$$

where the quantity $1-\frac{\omega_{p}^{2}}{\omega (\omega +i\gamma )}$ is just equal to ε(ω) mentioned in Equation (2). For an element $K_{e}$, we rewrite the local solution $\left(E_{h},H_{h}\right)$ and hybrid variable $\lambda_{h}$ like the form in Equation (17) of [17], i.e., at this moment, the discretized system is transformed to solve the following problem

(22) $$\left[\begin{array}{ccc} K_{ii}^{1} & 0 & K_{ig}^{1}\\ 0 & K_{ii}^{2} & K_{ig}^{2}\\ K_{gi}^{1} & K_{gi}^{2} & K_{gg}^{1}+K_{gg}^{2} \end{array}\right]\left[\begin{array}{c} \Lambda_{h,i}^{1}\\ \Lambda_{h,i}^{2}\\ \Lambda_{h,g} \end{array}\right]=\left[\begin{array}{c} b_{h,i}^{1}\\ b_{h,i}^{2}\\ b_{h,g}^{1}+b_{h,g}^{2} \end{array}\right],$$

where *g* represents the according degrees of freedom (DOFs) on $\Gamma_{1,2}$ and *i* indicates the according DOFs in $\Omega_{1}$ or $\Omega_{2}$. Moreover, this resulting linear system (22) is large, sparse but complex non-Hermitian, so the sparse direct solvers are always very expensive and prohibitive [26,27]. Thus, in the next section we use the Krylov subspace methods [21], which only depend on the information of the coefficient matrix-vector products.

Now we summarize the main steps of solving the Drude model with an optimized Schwarz method discretized by HDG method as follows:

- The model problem is split into some sub-problems with the corresponding subdomains which are discretized using an HDG method;
- Then we solve the resulting system of linear algebraic Equations (6) in each subdomain by a sparse direct solver;
- Finally, for the interface system between the two subdomains, solving the resulting linear systems (22) in the domain is accelerated using a Krylov subspace method.

## 5. Numerical Tests

In this section, we present two numerical results to show that the optimized Schwarz method is effective. All the numerical simulations are implemented in MATLAB R2012a and performed on a desktop with an AMD A6-6310 APU with AMD Radeon R4 Graphics CPU of 1.80 GHZ and 4.0 GB memory. We only employ the zeroth order approximation of $S_{l}$ in our tests. We use Gmsh (see https://gmsh.info/ (accessed on 15 September 2022)) to decompose the domains. In the following, “HDG-$P_{1}$” denotes the HDG discretization method relying on a nodal Lagrange basis interpolation of order p=1. For the numerical solution, we set the stopping criterion of the iteration process as $10^{-6}$; that is, when the relative residual

$$\frac{∥r^{k}{∥}_{2}}{∥r^{0}{∥}_{2}}<10^{-6},$$

then we stop the iteration of the Krylov subspace method, namely GMRES (DD-Gmres).

### 5.1. Cylindrical Nanowire Problem

**Figure 2:**
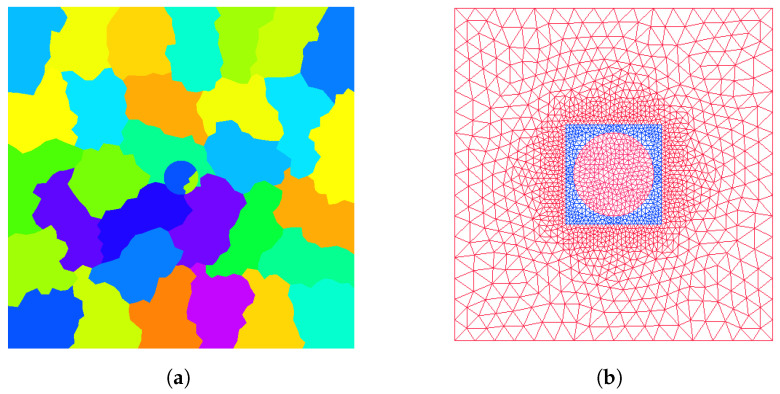
Cylindrical nanowire problem. (**a**) A subdomain decomposition and (**b**) meshes for the problem.

In this test, we set the radius of the cylinder to 20 nm. The interband transitions are ignored here. The computational domain $\Omega ={\left[-L,L\right]}^{2}$ is a square with L=200nm. We impose the artificial absorbing boundary condition on the boundary of Ω. In our test, we set $\omega_{p}=8.65\times 10^{15},\;\gamma =0.01\omega_{p}$ [28]. A typical subdomain decomposition is shown in Figure 2a. Meshes for the cylindrical nanowire problem are shown in Figure 2b.

In Table 2, we divide the domain into 3288 elements with 1645 nodes. In Table 3, we divide the domain into 13,024 elements with 6513 nodes. We chose $\omega =1.4\;\omega_{p}$ and $\omega =0.4\;\omega_{p}$ since they are closest to the resonance frequency of the material. In fact, the Maxwell’s equations are often considered to be more difficult to solve around the resonance frequency than other frequencies [5]. The results of different optimized parameters with different subdomains at $\omega =1.4\;\omega_{p}$ and $\omega =0.4\;\omega_{p}$ are presented in Table 2 and Table 3. The results show that the parameters with case3 do not work well for the Drude model problem. With the same number of subdomains, case1 and case2 converge much faster than the classical Schwarz method, and case3 which is consistent with Table 2 and Table 3. Furthermore, case1 outperforms case2 under the same number of subdomains. For each case, the number of iterations increases with the increasing of number of subdomains.

**Figure 3:**
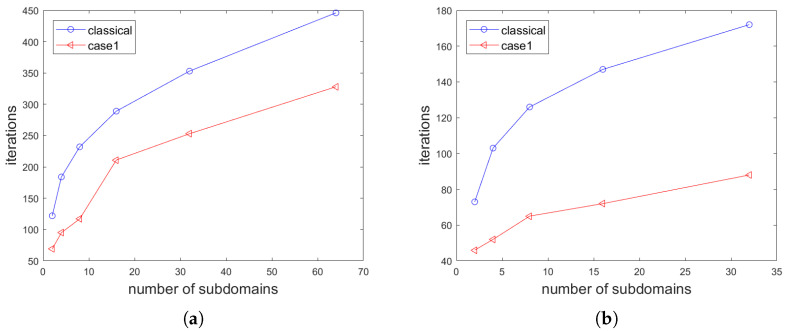
Number of GMRES iterations vs. number of subdomains. (**a**) $\omega =0.4\omega_{p}$ and (**b**) $\omega =1.4\omega_{p}$.

**Figure 4:**
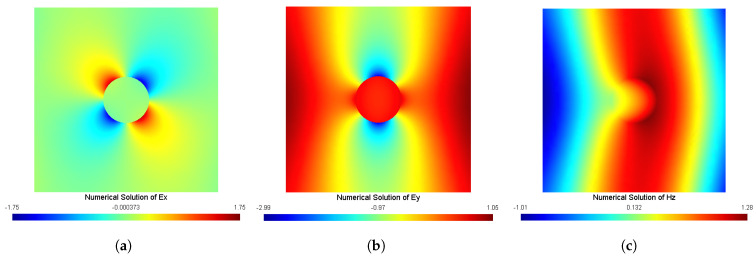
Field distributions of the cylindrical nanowire problem. (**a**) $E_{x}$, (**b**) $E_{y}$, and (**c**) $H_{z}$.

The domain in the following tests contains 13,024 elements with 6513 nodes. We show how the number of iterations required by the GMRES method varies with the number of subdomains at $\omega =1.4\omega_{p}$ and $\omega =0.4\omega_{p}$ in Figure 3. As seen from Figure 3, we can find that the number of iterations for GMRES increases with the number of subdomains. Additionally, case1 increases more slowly than that of the classical Schwarz method. For two subdomains, how the interpolation order *p* in the HDG formulation affects the convergence at the frequency $\omega =1.4\omega_{p}$ is shown in Table 4. We observe that the number of iterations increases with the increasing of *p* for each case. Furthermore, case1 outperforms the classical Schwarz method under the same *p*. Field distributions at the frequency $\omega =0.4\omega_{p}$ are presented in Figure 4. Note that in the above tests we set the value of the local stabilization parameter τ=1. The local stabilization parameter τ varies how it affects the number of iterations at $\omega =1.4\omega_{p}$; this is shown in Table 5.

### 5.2. Dimer of Cylindrical Nanowires

**Figure 5:**
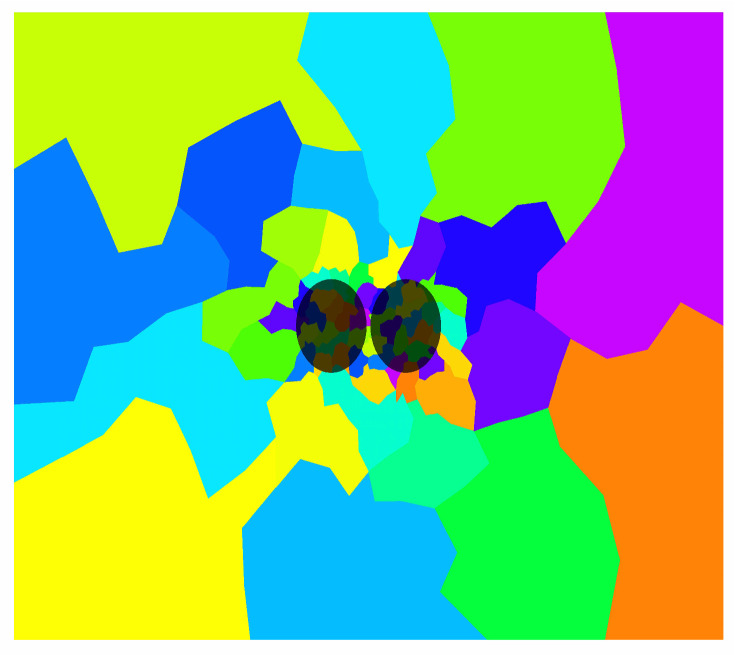
Dimer of cylindrical nanowires: a typical subdomain decomposition.

The computational domain is a rectangle with length and width of 300 nm and 200 nm, respectively. We divide the computational domain into 2226 elements with 1114 nodes. Then we consider the plasmonic dimer structures with small gaps [3,29]. We use the parameters in [3]: $\omega_{p}=1.34\times 10^{16},\gamma =1.14\times 10^{14}$. The radius of the cylinder is 30 nm. We present a typical subdomain decomposition in Figure 5.

**Figure 6:**
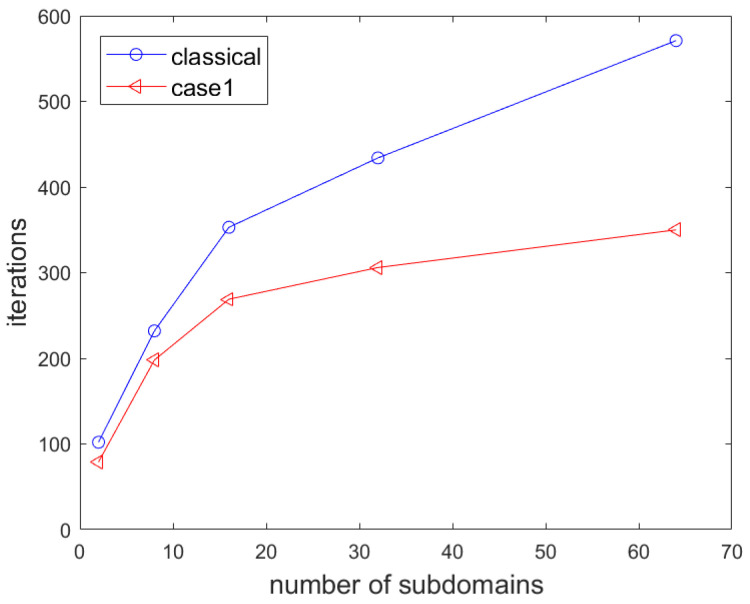
Number of GMRES iterations vs. number of subdomains.

**Figure 7:**
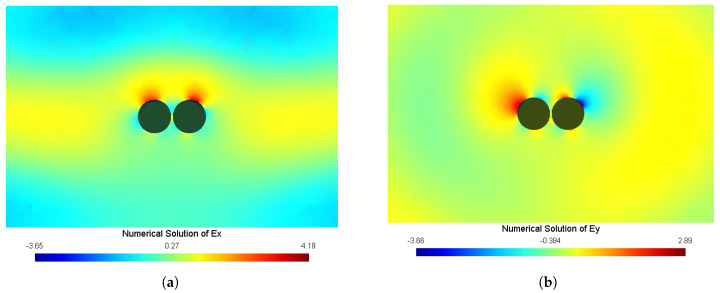
Field distributions of the dimer of cylindrical nanowires problem. (**a**) $E_{x}$ and (**b**) $E_{y}$.

Table 6 shows how the number of iterations varies with the number of subdomains at $\omega =0.4\;\omega_{p}$. As we can see from Table 6, case1 converges much faster than the classical Schwarz method with the same number of subdomains. For each case, the number of iterations increases as we increase the number of subdomains; how the number of iterations varies with the number of subdomains is shown in Figure 6. For two subdomains, how the interpolation order *p* in the HDG formulation affects the convergence is shown in Table 7. As seen from Table 7, we can find that the number of iterations increases as we increase the interpolation order. The field distributions at this particular frequency are displayed in Figure 7.

## 6. Conclusions

In the previous study [17], where the permittivity is a real number, the authors have solved the Maxwell’s equations with an optimized Schwarz method discretized by an HDG method, which performs well. In the current paper, the permittivity is a complex number in the Drude model, which adds the complexity to the optimized Schwarz method. We employ an optimized Schwarz method combined with an HDG discretization to solve the local optical response model. The domain is arbitrarily divided into several subdomains. New transmission parameters are proposed and tested. Numerical tests show that the optimized Schwarz method with a proposed parameter works quite well for Drude model problems.

For future work, it is noted that the coefficient matrix of the resulting linear system (22) is complex symmetric, which means that some particular Krylov subspace solvers [30,31] with suitable preconditioners for such linear systems can be employed to reduce the computational cost. In addition, it will be meaningful to extend the proposed method for solving three-dimensional model problems.

## Figures and Tables

**Table 1:** Spectral radius ρ and parameter α.

Cases	$\alpha_{l}$	ρ
classical	1	1
case1	${\left(i\omega \right)}^{\left(-1\right)}\left(-p_{l}+ip_{l}\right)$	0.6711
case2	${\left(i\omega \right)}^{\left(-1\right)}\left(-p_{l}-ip_{l}\right)$	0.9942
case3	${\left(i\omega \right)}^{\left(-1\right)}\left(p_{l}+ip_{l}\right)$	1.0059
case4	${\left(i\omega \right)}^{\left(-1\right)}\left(p_{l}-ip_{l}\right)$	1.4900

**Table 2:** The number of GMRES iterations for the cylindrical nanowire problem with HDG-$P_{1}$ (medium size discretization).

Nodes	Elements	Number of Subdomains		2	4	8
		Krylov Subspace Method		DD-Gmres	DD-Gmres	DD-Gmres
1645	3288	$\omega =0.4\omega_{p}$	case1	46	51	59
			case2	50	60	74
			case3	82	123	182
			classical	88	113	140
		$\omega =1.4\omega_{p}$	case1	39	43	50
			case2	53	67	86
			case3	100	169	267
			classical	65	83	100

**Table 3:** The number of GMRES iterations for the cylindrical nanowire problem with HDG-$P_{1}$ (large-scale discretization).

Nodes	Elements	Number of Subdomains		2	4	8
		Krylov Subspace Method		DD-Gmres	DD-Gmres	DD-Gmres
6513	13,024	$\omega =0.4\omega_{p}$	case1	69	95	117
			case2	73	104	131
			case3	111	189	293
			classical	122	184	232
		$\omega =1.4\omega_{p}$	case1	46	52	65
			case2	60	80	105
			case3	125	217	366
			classical	73	103	126

**Table 4:** The influence of interpolation order *p* in the HDG method on the number of GMRES iterations.

Interpolation Order	1	2	3
case1	46	56	65
case2	60	70	78
case3	125	137	149
classical	73	84	94

**Table 5:** The influence of the local stabilization parameter τ in the HDG method on the number of DD-gmres iterations.

Number of Subdomains	2				4			
**Value of** τ	τ=1	τ=−1	τ=i	τ=−i	τ=1	τ=−1	τ=i	τ=−i
case1	46	48	45	49	52	55	52	56
case2	60	58	57	61	80	81	79	83
case3	125	131	127	127	217	229	221	224
classical	73	85	78	84	103	119	107	120

**Table 6:** The number of GMRES iterations for the dimer of cylindrical nanowires with HDG-$P_{1}$.

Nodes	Elements	Number of Subdomains		16	32	64
		Krylov Subspace Method		DD-Gmres	DD-Gmres	DD-Gmres
1114	2226	$\omega =0.4\omega_{p}$	case1	269	306	350
			case2	278	317	397
			case3	400	508	632
			classical	353	434	571

**Table 7:** The influence of interpolation order *p* in the HDG method on the number of GMRES iterations.

Interpolation Order	1	2	3
case1	79	104	119
case2	82	108	120
case3	123	170	203
classical	102	137	157

## Data Availability

Data will be made available on request.

## References

[1] Sattler K.D. (2010). Handbook of Nanophysics: Nanoelectronics and Nanophotonics.

[2] Maier S.A. (2007). Plasmonics: Fundamentals and Applications.

[3] Li L., Lanteri S., Mortensen N.A., Wubs M. (2017). A hybridizable discontinuous Galerkin method for solving nonlocal optical response models. Comput. Phys. Commun..

[4] Raza S., Bozhevolnyi S.I., Wubs M., Mortensen N.A. (2015). Nonlocal optical response in metallic nanostructures. J. Phys. Condens. Matter.

[5] Mortensen N.A., Raza S., Wubs M., Bozhevolnyi S.I., Søndergaard T. (2014). A generalized non-local optical response theory for plasmonic nanostructures. Nature Commun..

[6] Chaumont-Frelet T., Lanteri S., Vega P. (2021). A posteriori error estimates for finite element discretizations of time-harmonic Maxwell’s equations coupled with a non-local hydrodynamic Drude model. Comput. Methods Appl. Mech. Eng..

[7] Aeschlimann M., Brixner T., Fischer A., Hensen M., Huber B., Kilbane D., Kramer C., Pfeiffer W., Piecuch M., Thielen P. (2016). Determination of local optical response functions of nanostructures with increasing complexity by using single and coupled Lorentzian oscillator models. Appl. Phys. B.

[8] Sun B., Ji B., Lang P., Qin Y., Lin J. (2022). Local near-field optical response of gold nanohole excited by propagating plasmonic excitations. Opt. Commun..

[9] Wen S.-S., Tian M., Yang H., Xie S.-J., Wang X.-Y., Li Y., Liu J., Peng J.-Z., Deng K., Zhao H.-P. (2021). Effect of spatially nonlocal versus local optical response of a gold nanorod on modification of the spontaneous emission. Chin. Phys. B.

[10] Hesthaven J.S., Warburton T. (2007). Nodal Discontinuous Galerkin Methods: Algorithms, Analysis, and Applications.

[11] Arnold D.N., Brezzi F., Cockburn B., Marini L.D. (2002). Unified analysis of discontinuous Galerkin methods for elliptic problems. SIAM J. Numer. Anal..

[12] Dolean V., Fol H., Lanteri S., Perrussel R. (2008). Solution of the time-harmonic Maxwell equations using discontinuous Galerkin methods. J. Comput. Appl. Math..

[13] Monk P. (2003). Finite Elementlement Methods for Maxwell’s Equations.

[14] Cockburn B., Gopalakrishnan J., Lazarov R. (2009). Unified hybridization of discontinuous Galerkin, mixed, and continuous Galerkin methods for second order elliptic problems. SIAM J. Numer. Anal..

[15] Nguyen N.C., Peraire J., Cockburn B. (2011). Hybridizable discontinuous Galerkin methods for the time-harmonic Maxwell’s equations. J. Comput. Phys..

[16] Li L., Lanteri S., Perrussel R. (2013). Numerical investigation of a high order hybridizable discontinuous Galerkin method for 2d time-harmonic Maxwell’s equations. Compel-Int. J. Comp. Math. Electr. Electron. Eng..

[17] He Y.-X., Li L., Lanteri S., Huang T.-Z. (2016). Optimized Schwarz algorithms for solving time-harmonic Maxwell’s equations discretized by a hybridizable discontinuous Galerkin method. Comput. Phys. Commun..

[18] Bouajaji M.E., Dolean V., Gander M.J., Lanteri S. (2012). Optimized Schwarz methods for the time-harmonic Maxwell equations with damping. SIAM J. Sci. Comput..

[19] Dolean V., Lanteri S., Perrussel R. (2008). Optimized Schwarz algorithms for solving time-harmonic Maxwell’s equations discretized by a discontinuous Galerkin method. IEEE Trans. Mag..

[20] Li L., Lanteri S., Perrussel R. (2014). A hybridizable discontinuous Galerkin method combined to a Schwarz algorithm for the solution of 3d time-harmonic Maxwell’s equation. J. Comput. Phys..

[21] Saad Y. (2003). Iterative Methods for Sparse Linear Systems.

[22] Kong J.A. (1986). Electromagnetic Wave Theory.

[23] Li K., Huang T.-Z., Li L., Lanteri S. (2023). Simulation of the interaction of light with 3-D metallic nanostructures using a proper orthogonal decomposition-Galerkin reduced-order discontinuous Galerkin time-domain method. Numer. Methods Partial Differ. Equ..

[24] Dolean V., Gander M.J., Gerardo-Giorda L. (2009). Optimized Schwarz methods for Maxwell’s equations. SIAM J. Sci. Comput..

[25] Gander M.J. (2006). Optimized Schwarz methods. SIAM J. Numer. Anal..

[26] Bollhöfer M., Schenk O., Janalik R., Hamm S., Gullapalli K., Grama A., Sameh A.H. (2020). State-of-the-art sparse direct solvers. Parallel Algorithms in Computational Science and Engineering.

[27] Gu X.-M., Zhao Y., Huang T.-Z., Zhao R. (2021). Efficient preconditioned iterative linear solvers for 3-D magnetostatic problems using edge elements. Adv. Appl. Math. Mech..

[28] Hiremath K.R., Zschiedrich L., Schmidt F. (2012). Numerical solution of nonlocal hydrodynamic drude model for arbitrary shaped nano-plasmonic structures using Nédélec finite elements. J. Comput. Phys..

[29] Toscano G., Raza S., Jauho A.-P., Mortensen N.A., Wubs M. (2012). Modified field enhancement and extinction by plasmonic nanowire dimers due to nonlocal response. Opt. Express.

[30] Gu X.-M., Huang T.-Z., Li L., Li H.-B., Sogabe T., Clemens M. (2014). Quasi-minimal residual variants of the COCG and COCR methods for complex symmetric linear systems in electromagnetic simulations. IEEE Trans. Microw. Theory Techn..

[31] Gu X.-M., Clemens M., Huang T.-Z., Li L. (2015). The SCBiCG class of algorithms for complex symmetric linear systems with applications in several electromagnetic model problems. Comput. Phys. Commun..

